# Combining public health evidence, policy experience and communications expertise to inform preventive health: reflections on a novel method of knowledge synthesis

**DOI:** 10.1186/s12961-023-01062-x

**Published:** 2023-10-31

**Authors:** Maddie Heenan, Alexandra Chung, Elly Howse, Helen Signy, Lucie Rychetnik

**Affiliations:** 1 The Australian Prevention Partnership Centre, Sax Institute, Sydney, Australia; 2 The George Institute for Global Health, University of New South Wales, Sydney, Australia; 3 School of Public Health and Preventive Medicine, Monash University, Melbourne, Australia; 4 School of Public Health, University of Sydney, Sydney, Australia

**Keywords:** Knowledge translation, Evidence synthesis, Prevention, Coproduction

## Abstract

Knowledge synthesis methods help summarize evidence and utilize content expertise to draw out key messages to aid knowledge mobilization and translation. Systems thinking and coproduction can support this by facilitating a multiperspective view and ensuring that knowledge is mobilized and translated in a useful and meaningful way for policy-makers and practitioners. In this paper, we describe the development of a knowledge synthesis approach that utilizes coproduction with policy-makers to combine the findings of a programme of research with policy knowledge to support decision-makers working in chronic disease prevention. The process developed by The Australian Prevention Partnership Centre combined the expertise of research, policy and science communications experts. We reflect on how we used coproduction processes to embed policy-makers as partners in the evidence synthesis process via research-policy dialogues, and embedded science communication into the development and presentation of the findings. This differs from a more common approach of researchers generating evidence for policy with limited input from policy-makers themselves. By collaborating with policy-makers and using coproduction, we can better inform policy-relevant research and generate policy-relevant knowledge. We describe the development of our knowledge synthesis approach using two case studies: the first drawing on a body of work in public health law, and the second on a body of work focused on the first 2000 days of life. We consider how these case studies demonstrate the value of working with policy partners as part of a knowledge synthesis process, and discuss how this process could be adapted and used in future.

## Introduction

Preventive health research generates an abundance of new knowledge and empirical evidence on what works to support and promote improved population health. However, the health problems that this research seeks to address are complex challenges with multiple causes and solutions, and there continues to be slow progress in many areas; for example, despite prevention efforts obesity rates continue to rise [1]. Much of the research literature on chronic disease prevention is still occupied with describing problems [2, 3] rather than implementing, evaluating and scaling-up solutions to the level of systems change needed to improve population health [4–6].

One approach to dealing with the ongoing challenges of evidence-informed decision-making is a knowledge synthesis process, recognized as a valuable mechanism to help policy-makers and practitioners apply and use evidence [7, 8]. Specific research methodologies are commonly used including systematic reviews and meta-analyses [9, 10]. However, these methods often rely heavily on quantitative evidence and research questions predominantly derived from researchers. Researchers are encouraged to articulate the applied policy and practice implications of their work [11, 12], and this is made meaningful for decision-makers when it is considered in the context of related bodies of work, other literature and their own priorities [13]. Yet, reviews of evidence are most useful for policy-makers when there is input from policy-makers themselves [14, 15]. Embedding participatory codesign or coproduction processes into knowledge synthesis methods can help enhance effective science communication and ensure that knowledge and evidence is relevant, user friendly and directly available to the end users and decision-makers. However, there is currently little guidance about approaches to synthesizing evidence and knowledge that focus on combining scientific evidence with policy-maker knowledge via coproduction, then translating this into key messages and narratives that can be more readily used by decision-makers.

In this paper, we describe a policy-relevant knowledge synthesis method that addresses this gap. We reflect on how coproduction processes were used to synthesize learnings from a body of research and embed policy-maker and science communication perspectives into a new type of evidence synthesis process. This builds on previous research that reviews of evidence for policy-makers are more useful with input from the policy-makers as end users [14, 15]. It also builds on the extensive experience in research translation and science communication of The Australian Prevention Partnership Centre (Prevention Centre) [16–18]. In this paper, we describe and outline the rationale for this policy-relevant knowledge synthesis approach, the theories that informed its development and present two case studies of how the process was used and adapted in practice.

### Contextualizing the knowledge synthesis

The Prevention Centre is a collaboration of policy-makers, researchers and practitioners, working together to improve the availability and value of Australian policy-relevant research on the prevention of chronic disease [16]. It has been cofunded by Australia’s National Health and Medical Research Council (NHMRC), Commonwealth, state and territory health departments, and nongovernment agencies, and its work is underpinned by systems thinking and coproduction [17, 18].

Since its inception in 2013, the Prevention Centre has supported over 70 research projects and led innovative work in applying systems approaches to knowledge mobilization [19]. In 2020, the Prevention Centre also established the Collaboration for Enhanced Research Impact (CERI), a joint initiative with a growing number of NHMRC Centres of Research Excellence (CREs) that aims to identify and implement new ways to mobilize policy-relevant prevention research [20]. While most discrete projects provide valuable findings on their given research questions, policy partners are primarily interested in the practical implications of larger bodies of research. The knowledge synthesis process reported here was developed to derive the learnings and insights from across our programmes of work, and to facilitate engagement with our policy partners to frame the synthesis questions and collaborate on interpreting the findings.

Public health law and the first 2000 days of life were identified by the Prevention Centre’s funding partners as two priority topics for programme-wide knowledge synthesis. In developing this process, we were guided by three strategic questions:

1. What can we learn from the findings to date of Prevention Centre-funded and CERI-supported research when we consider the findings across the whole body of work?
2. How do these insights add to the existing body of evidence on this topic?
3. What are the implications for Australian research, policy and/or practice?

Our knowledge synthesis process was first developed using the body of work supported through the Prevention Centre on the topic of public health law for prevention, and then developed further using work drawn from the Prevention Centre and CERI projects on the topic of the first 2000 days of life. The synthesis findings and related resources are presented online in accessible formats [21].

### Applying coproduction and systems thinking

Knowledge synthesis methods help summarize evidence and expertise, and draw out key messages from diverse research to help facilitate knowledge mobilization or translation [22, 23]. Knowledge translation and knowledge mobilization are terms often used interchangeably. Other similar variations are also found in the literature (for example, knowledge transfer, knowledge exchange, knowledge integration) and while there are differences in approach, they are all variations of knowledge mobilization, aiming to create, share and apply knowledge [7]. Our interpretation of knowledge mobilization is that it attempts to support meaningful usage of evidence and expertise to align research, policy and practice to improve health outcomes.

Applying systems thinking to knowledge mobilization in public health facilitates a multiperspective view on complex phenomena such as public health and prevention [19, 24]. Systems thinking posits that interrelated but independent parts are linked within a complex system, and can have direct and indirect influence on situations [25, 26]. Applying a systems lens is fundamental to addressing social determinants of health and complex public health issues [1, 5].

Knowledge synthesis is an important component of knowledge mobilization. While only a part of the mobilization process, knowledge synthesis also benefits from incorporating systems approaches to inform how you examine and understand the context in which you work and by incorporating multiple perspectives [27, 28]. This can be supported by coproduction, which facilitates collaborative approaches and contextual understanding. It is a mechanism where stakeholders collaborate, generate knowledge that is relevant to the context and apply it in practice [29, 30]. Similar to the knowledge mobilization literature, different variations of coproduction (for example, codesign, cocreate, coproduce) are used; however, “co” is suggestive of the collaborative or participatory design [31]. By involving key stakeholders, coproduction is likely to improve the impact and feasibility of a process, as you are involving the target audience or “end user” [18].

## The knowledge synthesis process

Our knowledge synthesis process combined the expertise of research, policy and communications practitioners, and drew on systems thinking and principles of knowledge mobilization by embedding multiperspectivity and coproduction. We proposed that conducting a knowledge synthesis in partnership with policy-makers would enhance the relevance and responsiveness to an unmet need for evidence, and increase the impact of the research conducted through the Prevention Centre and its CERI partners.

In developing this process, our objectives were to:

1. Identify priority areas of research and policy that are of interest to our funding partners
2. Synthesize the evidence, knowledge and expertise from a given priority area
3. Translate the synthesis findings into a series of outputs that integrate research, communications and policy expertise
4. Share these outputs with policy partners and the broader prevention system to inform policy considerations and discussions on systems change

**Fig. 1:**
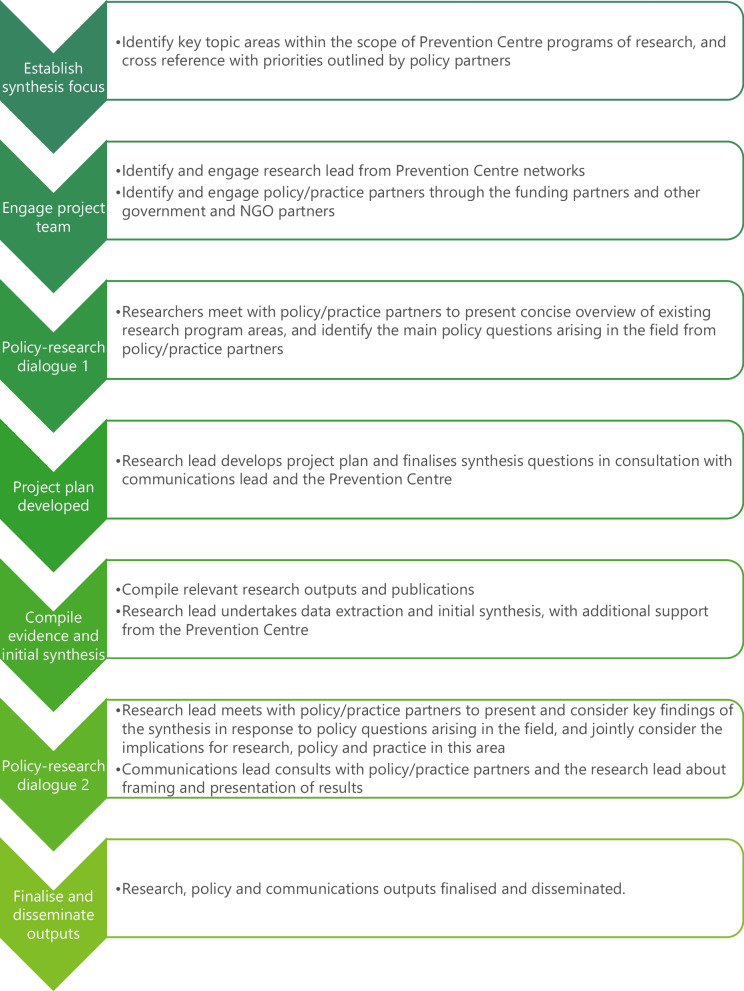
The Australian Prevention Partnership Centre knowledge synthesis process

Each of the two knowledge syntheses reported here focused on a selective body of research and involved a research content lead, a communications lead and self-selected policy partners with interest in the topic area. Research-policy dialogues were held to engage policy partners and coproduce the questions to be addressed by the synthesis and the interpretation of the results. The communications lead also assisted with framing and communication throughout the knowledge synthesis process. The dialogues were held online via videoconferencing platform. They were of 90–120 min duration, with discussions occurring verbally and in the chat function. Dialogues were facilitated by an individual with policy and research experience, thereby providing them with an understanding of both groups and the ability to draw out mutual objectives. Framing the knowledge synthesis included reflecting key questions back to the group during the meeting, as well as after in written form, to ensure these reflected the discussions and were understood and agreed by the participants. The second research-policy dialogue invited the policy partners to discuss the policy implications of the synthesized findings to help frame the final reporting and associated communications. The steps undertaken throughout the knowledge synthesis process are outlined in Fig. 1 and the application of the process in the two case studies is discussed in more detail below.

### Communicating the science

One key part of this novel knowledge synthesis process was the involvement from the outset of science communication professionals. The primary aim of science communication is to find the most effective ways to communicate complex information to people outside the scientific research arena [32]. This reflects a growing recognition that effective translation of research findings needs to apply science communications expertise and the “use of the appropriate skills, media activities and dialogue” to enhance research impact [33, 34]. In this process, our aim was to further develop conventional models of science communication by incorporating a more participatory model that would support the synthesis process and inform the outputs.

The research-policy dialogues included discussion of how the end-users would like to receive the knowledge synthesis findings, including framing of messages and what communication products would suit their practical needs. Findings were disseminated in two reports, with infographics and other design elements to improve understanding and accessibility for policy-makers. Other knowledge products were produced, including evidence summaries, a policy brief and two episodes of the “Prevention Works” podcast. All the products were published on the Prevention Centre website and disseminated using tailored communication to achieve maximum reach among the target audiences [21].

### Applied case studies: synthesized work in public health law and the first 2000 days

The knowledge synthesis process was first developed with the public health law case study. It focused on scoping the diverse range of study topics encompassed in the field of public health law and defining the nature and boundaries of that body of work. Policy colleagues contributed to these early scoping discussions, and the research-policy dialogues were held half-way and towards the end of the knowledge synthesis process. For the second case study, the knowledge synthesis process was reflexively updated to include a research-policy dialogue at the beginning of the process, and again at the end of the draft synthesis. The second case study focused on prevention in the first 2000 days of life, which was an established programme of research with clearer boundaries of what is in scope. The research-policy dialogues helped to define the review questions, and informed the conclusions about the interventions examined. Each of these case studies are described in more detail below. Ethics approval was not sought for this work. The syntheses examined published literature and did not involve collecting new data. The research-policy dialogues were conducted with existing partners to guide the questions and conclusions of the syntheses.

#### Case study 1: public health law

Public health law is a well-established and important component of effective chronic disease prevention. However, public health law research is a relatively new field of research [35]. Research in public health law looks at the determinants and relationships between law, policy-making and health. Research on specific topics is common, for example food labelling laws and implications for diet and obesity prevention, yet little work has been undertaken to synthesize the evidence across areas of public health law and in dialogue with policy decision-makers. This knowledge synthesis aimed to fill that gap and inform discussions on future research.

Our knowledge synthesis purposefully sampled projects and studies that related to law and regulation from the Prevention Centre’s funded research from 2013 to 2021 [36]. This included 12 projects and 40 publications. We identified Prevention Centre projects that had either a focus on specific issues in law and regulation for prevention, or “big policy” issues that set the regulatory agenda. Peer-reviewed literature, reports and communications material from relevant projects were assessed individually for inclusion. We analysed project outputs by public health topic, jurisdiction and research focus, and then thematically analysed the findings of the research to identify common themes across public health law.

Two research-policy dialogues were held with the Prevention Centre’s policy partners to define the framing and scope of the analysis, and to consider the policy and practice implications of the synthesis findings. However, as noted, the first research-policy dialogue was held well into the synthesis process of the first case study. As a result, this dialogue gathered policy partners’ feedback on the scoping already conducted, and the value of public health law research more generally. The definitions of law, regulation and policy were also discussed and refined during the dialogue to help further guide the synthesis. A nuanced approach was adopted that expressed the difference between policy as a strategy, and law and regulation as the tools to implement policy. The dialogues thereby led us to more clearly articulate the range of approaches available to policy-makers and to scope options for researchers studying the field. This synthesis led us to reflect on the availability and relevance of evidence for different aspects of our partners’ work that is reliant on public health law. We concluded that different types of evidence are needed at different stages of the policy and regulation process (for example, epidemiological evidence, regulatory analysis, impact evaluations) and can have different purposes for policy design and implementation. The second dialogue in the first case study discussed the knowledge synthesis findings and how these findings related to the everyday practice of our policy partners.

The knowledge synthesis identified five themes in the body of work on public health law: monitoring and evaluation; political environment considerations; regulatory design, implementation and enforcement; engagement, collaboration and coproduction; and impact on equity and disadvantage. The overlapping nature of some themes facilitated cross-cutting discussion, in particular the linkages between evaluating health outcomes and monitoring public health law; the overlap of regulatory design and enforcement with gaps and failures; and the relationships between the political environment, differing portfolio objectives and industry influence.

Some key reflections identified through this synthesis were as follows:

1. Public health law can provide the rules and frameworks to shape social and commercial determinants of health
2. Public health law research can help make the case for investment in prevention
3. Independent and coproduced research both make different contributions to public health law
4. There are opportunities for public health law to strengthen a cobenefits approach across health and other sectors

This synthesis strengthened the conceptual and practical linkages between public health research and practice. Research on law, regulation and policy for prevention is about communicating the value of the sometimes small but often powerful changes that law and regulation can produce to achieve a policy objective for effective, equitable prevention. Outputs arising from this synthesis included a report, evidence summary and podcast episode [21].

#### Case study 2: first 2000 days

The first 2000 days were defined as the period of early life from conception in pregnancy to age 5 years. This is a critical stage during which lifelong trajectories of health and well-being can be established, and presents an opportunity for preventive health interventions to promote and support health and reduce disease risk. The importance of the early life period is recognized in Australia with the National Preventive Health Strategy articulating a focus on giving every child the best start in life through promoting health and prevention of chronic disease risk factors from the prenatal period throughout childhood [37].

With the first 2000 days also identified as a priority area within CERI, this knowledge synthesis aimed to generate collective insights from CERI member Centres of Research Excellence (CREs) (six at the time of conducting the synthesis) and Prevention Centre projects, with engagement from policy partners to consider the implications for research, policy and practice [38].

Policy and practice partners from existing networks were invited to the first of two research-policy dialogues to identify policy priorities within the first 2000 days and develop policy-relevant questions to guide the knowledge synthesis. During this meeting, policy partners helped define the framing and scope of the synthesis and highlighted their evidence needs to progress policy and practice in the first 2000 days. From this dialogue, the following synthesis questions were developed:

1. What is the evidence for the benefits of prevention in the first 2000 days?
2. What prevention interventions are effective (and cost-effective) to give children the best start in life?
3. How do we support implementation and scale-up of effective interventions?
4. How can we tailor, implement and scale-up prevention interventions to meet the needs of priority population groups including Aboriginal and Torres Strait Islander peoples, culturally and linguistically diverse communities, and people experiencing socioeconomic disadvantage?

Relevant articles were sought from research partners across CERI member CREs and from Prevention Centre projects. Of 78 articles reviewed, 60 were deemed to be relevant to prevention in the first 2000 days and subsequently included in the initial synthesis.

Findings from the synthesized research evidence were presented to policy partners in the second of two research-policy dialogues where implications for policy and practice were discussed. Key findings drawn through the synthesis process included:

- There is a window of opportunity to establish and support healthy behaviours in the formative first 2000 days of life.
- There is strong public support for prevention interventions in the first 2000 days, particularly for interventions that protect children’s health.
- Investing in prevention in the first 2000 days yields economic benefits.
- Healthy lifestyle interventions during preconception and pregnancy, and family-based early childhood obesity prevention interventions have demonstrated evidence of effectiveness and cost-effectiveness.
- Prevention in the first 2000 days requires a comprehensive approach that combines individual and population-based interventions to support healthy behaviours, promote health in settings (including education, workplace and healthcare settings), and create healthy and supportive environments.
- Implementation and scale-up of effective interventions requires collaboration between researchers, policy-makers, practitioners and consumers, and a careful balance of programme fidelity and tailoring to local need.

Outputs arising from this knowledge synthesis include a report outlining the findings in detail as well as a policy brief and a podcast episode to disseminate key evidence in several accessible formats [21].

## Reflections and discussion

This paper describes and reflects on the development of a process for knowledge synthesis and translation which embeds policy-makers and communications experts into the synthesis process, and utilizes their expertise to ensure outputs are policy relevant and shared in engaging and meaningful ways. It goes beyond traditional knowledge synthesis methods, such as systematic reviews, by coproducing the research questions, integrating knowledge directly from policy-makers and jointly considering the implications of the findings in practice. The process supported policy-makers and researchers to discuss the scope and findings of a body of research and consider the real-world implications. Substantial investments in knowledge production and evidence generation have historically been under-utilized [12]. In the context of public health policy-making, there is often a dual problem of not enough of the right type of evidence available at the time it is required, and too much evidence being generated for time constrained policy-makers to digest and interpret. There is also the issue of evidence syntheses being disconnected from policy processes. For example, syntheses that are not policy relevant or so independent that policy-makers are unaware of the findings, as well as limited connections or opportunities for connection between researchers and policy-makers. Our novel approach fills this gap. Reflecting on two distinct case studies, we suggest our knowledge synthesis process helped to identify, prioritise and respond to policy-makers’ needs for synthesized and interpreted evidence. We also suggest it promoted connection and engagement among academic researchers and policy-makers and practitioners, particularly at the early- to mid-career level, which we believe is vital for promoting systems change for prevention.

Our method aimed to generate a process for knowledge synthesis that answers policy-relevant questions via an engaging, user-friendly format, and can be updated as new knowledge and evidence comes to light. During the final research-policy dialogues, we reflected with policy partners on the framing of key messages and identified which communication products would best suit their practical needs. As a result, summary briefs were developed in conjunction with a longer report, which was supported by other media such as podcasts. By tailoring communication products directly to meet the stated needs of interested policy-makers nationally, the Prevention Centre aimed to expedite the value and eventual impact of the synthesized findings. These resources have been disseminated to the policy partners engaged in the process, as well as through our website and broader communications.

In the 9 months following the publication of the public health law knowledge synthesis, there have been 51 downloads of the full report and 57 downloads of the summary brief. In the 12 months following the publication of the first 2000 days knowledge synthesis, there have been over 400 downloads of the report and over 450 downloads of the summary brief. The first 2000 days knowledge synthesis also developed a policy brief which has seen 420 downloads in the past 12 months. Anecdotally, our policy partners have informed us that the knowledge syntheses have been useful and widely shared among public health policy and practice colleagues.

Another value of our approach in utilizing research-policy dialogues is that policy-makers can become better informed of the evidence as it is being synthesized, as well as research that is underway or has been recently completed. Real-time, targeted information can be provided to support their work as opportunities emerge. This is compared with large systematic reviews and slower-moving peer-review publications.

The application of the process across projects in different areas of chronic disease prevention helped us to develop and refine a novel knowledge synthesis approach and examine the versatility of the process. We note the later timing of the first research-policy dialogue in case study one, which was more conceptual and focused on mapping the scope and boundaries of the research. While this focus partly reflected the nature of the topic being reviewed, we suggest that for future knowledge syntheses a scoping research-policy dialogue is always included early in the process. Since the first two case studies were conducted, two more knowledge syntheses are in progress on the topics of health economics and implementation science. These also include research-policy dialogues at the start and towards the end of the synthesis.

We are continuing to refine the dialogue process. For example, to provide more opportunity for engagement and discussion, in addition to the verbal communication and chat functions in the video conference, the new knowledge syntheses will employ Padlet, an online platform where additional written feedback can be added in real time or over several days post dialogue. Contributions can be made anonymously or not, providing more opportunity and different forms of engagement for situations where individuals or ideas may dominate verbal discussions, sensitive information cannot be disclosed to all participants and/or additional thoughts occur post discussion. While the use of research-policy dialogues in the knowledge synthesis process will continue to evolve, another important next step is to evaluate how policy-makers are using the knowledge products that have been developed, and evaluate and appraise the utility of the products against policy partners’ needs.

The Prevention Centre is tasked by its funding partners to develop new methods of policy-relevant evidence synthesis. We have done this previously through the development of participatory dynamic simulation modelling, a style of knowledge synthesis that combines diverse forms of evidence and participatory dialogues [39, 40]. We developed the narrative form of policy-relevant knowledge synthesis described in this paper in dialogue with our partners. It seeks to draw out the lessons from our existing body of work by drawing on the research, the policy expertise of our partners and science communication expertise to create new, user-friendly resources that could inform policy-making. It is important to note that the knowledge synthesis examples presented in this paper are not typical systematic reviews. The evidence and knowledge focused on the work led by the Prevention Centre and its CERI partners, and applied the expertise of our policy partners to frame the questions and interpret the findings. By using policy partner perspectives to inform and frame research questions, experiential knowledge and insight was able to be integrated that researchers alone do not have and may otherwise have been overlooked. While the synthesis findings are considered in the context of the wider published literature, they do not claim to be, nor are meant to replace systematic reviews of all the evidence published in the field. Instead, they offer a practical, evidence-based and experiential approach to synthesizing knowledge, framing key messages, and developing and disseminating communication products that are fit-for-purpose for policy-makers and that can complement the evidence found in systematic reviews.

## Conclusions

This knowledge synthesis method has been developed to explore options for addressing some of the issues and challenges relating to policy-relevant synthesis of research and evidence. By collaborating with policy-makers and using coproduction, we can generate more policy-relevant knowledge and support future policy-relevant research. Additionally, by better understanding the needs and constraints of policy-makers, and the most useful formats in which they prefer to digest knowledge and evidence, we can better support policy partners in their decision-making.

## Data Availability

Not applicable—no new data was collected.
